# *Rhodotorula* Endogenous Endophthalmitis: A Novel Harbinger of the Injection Drug Epidemic in the United States

**DOI:** 10.1155/2017/9686353

**Published:** 2017-04-05

**Authors:** Preston M. Luong, Basilio Kalpakian, Lawrence J. Jaeger, Timothy Lahey, Christopher B. Chapman, Michael E. Zegans

**Affiliations:** ^1^Geisel School of Medicine at Dartmouth, Hanover, NH, USA; ^2^Section of Ophthalmology, Dartmouth-Hitchcock Keene, Keene, NH, USA; ^3^Section of Infectious Disease, Department of Medicine, Dartmouth-Hitchcock Medical Center, Lebanon, NH, USA; ^4^Section of Ophthalmology, Department of Surgery, Dartmouth-Hitchcock Medical Center, Lebanon, NH, USA

## Abstract

Endogenous endophthalmitis is a rare but feared infectious ocular complication of injection drug use (IDU). The recent opioid epidemic in the United States threatens to increase the incidence of this disease. We report the first case of endogenous endophthalmitis in the United States caused by the emerging fungal pathogen* Rhodotorula* in an injection drug user which led to no light perception vision (NLP). Worldwide experience with* Rhodotorula* endogenous endophthalmitis is limited, but existing cases suggest infection by this particular fungal genus has a grim prognosis.

## 1. Introduction

Mortality from the United States opioid epidemic has tripled to 9 per 100,000 persons since 2000 [1]. The health problems associated with injection drug use (IDU) are legion, with endogenous endophthalmitis among the most debilitating. Endogenous endophthalmitis can result from blood stream dissemination of organisms that contaminate injected drugs and may occur alone or secondary to endocarditis [2].

Fungi and bacteria both cause endogenous endophthalmitis with the most common pathogens being streptococci, staphylococci, and* Candida* spp. [2]. The rise of IDU raises the opportunity for uncommon microbes to be implicated as causative agents.* Rhodotorula* is a pink-pigmented yeast found widely in soil, water, and air. It has been isolated in infections from catheters and other foreign bodies [3]. A 2008 systematic review of all* Rhodotorula* infections in the medical literature cited only two cases of* Rhodotorula* endogenous endophthalmitis worldwide [3]. To our knowledge, no other case has been reported since. Here we present the first described case of* Rhodotorula*-associated endogenous endophthalmitis in the United States.

## 2. Case Report

A 21-year-old man with four days of left sided vision loss, eye pain, and conjunctival injection was examined. He reported floaters for two weeks. He was previously healthy but admitted to IDU. He had no recent surgeries, dental work, or intravenous catheters.

Upon examination in the left eye, the best-corrected visual acuity was counting fingers at one foot. Slit lamp examination revealed 4/4 conjunctival injection, 4/4 anterior chamber cells with hypopyon, and posterior synechiae. Ophthalmoscopic exam yielded a hazy view of two white collections in the posterior vitreous. The right eye was normal upon examination. He underwent pars plana vitrectomy with intravitreal injection of amphotericin B (5 mcg), ceftazidime (2.25 mg), and vancomycin (1.0 mg). He was discharged to home with atropine (1%), moxifloxacin (0.5%), prednisolone (1%), and oral azithromycin (250 mg). No microbes were isolated from blood and vitreous cultures nor did Gram stain or calcofluor white reveal any organisms.

**Figure 1:**
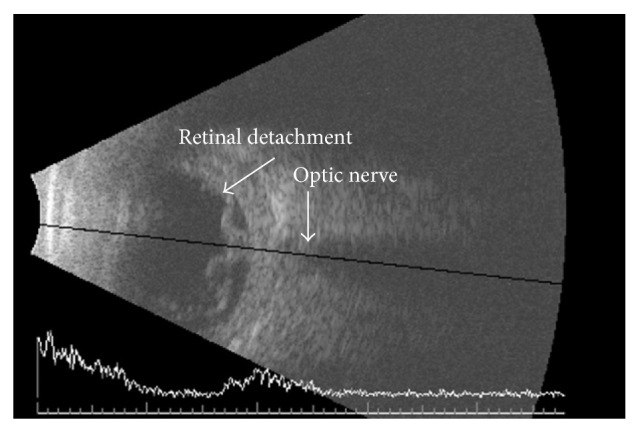
OS B-scan 10 days after final vitrectomy demonstrating funnel retinal detachment tethered to optic nerve head.

At one-month follow-up, although pain had completely subsided, visual acuity remained unchanged at hand motion along with nonclearing vitritis and a posterior pole white mass suggestive of an abscess. A second pars plana vitrectomy with abscess debridement and intravitreal injections of ceftazidime (2.25 mg) and vancomycin (1.0 mg) was performed. He was again discharged with moxifloxacin (0.5%) and prednisolone (1%) drops. A culture of the abscess identified colonies of the* Rhodotorula* species susceptible to amphotericin B and posaconazole. The specific species was narrowed to either* R. mucilaginosa* or* R. glutinis* through observation of morphology, pigmentation, and biochemical testing via the BioMerieux Vitek II Yeast Identification Card. Oral posaconazole (800 mg) was prescribed for 14 days. Unfortunately, a funnel retinal detachment developed 10 days after surgery (Figure 1). This detachment was deemed inoperable by two retinal specialists because of extensive retinal ischemia. No clinical recurrence of infection occurred however. His most recent follow-up visit three years later revealed no light perception in a left phthisis bulbi.

Table 1 relates the present case, the only report of* Rhodotorula* endogenous endophthalmitis in the United States, to the two other known cases [4, 5].

## 3. Discussion

The visual prognosis is poor in the limited worldwide clinical experience with* Rhodotorula *endogenous endophthalmitis: all three known cases including ours were associated with IDU and resulted in worse than light perception vision. The most common fungal endogenous endophthalmitis, caused by* C. albicans*, rarely results in visual loss of this severity [2]. The prognosis of* Rhodotorula* endogenous endophthalmitis stands in contrast to the better prognoses seen in other eye infections such as scleritis and keratitis caused by this rare yeast [6, 7]. Although vitreous culture is the gold standard for the diagnosis of fungal endogenous endophthalmitis, its sensitivity varies greatly, with values reported as low as 40% [8]. Our case underlines the importance of resampling and retreating eyes with suspected endogenous endophthalmitis as microbes may be sequestered and inaccessible in an abscess.

Our patient is the first described case of* Rhodotorula* endogenous endophthalmitis in the United States. Increasing opioid use in the last decade portends a rise of endogenous endophthalmitis and the emergence of rare infectious agents and poor visual outcomes.* Rhodotorula* should be considered in the differential diagnosis of IDU patients with sudden profound visual loss, especially if response to initial therapy is suboptimal. Given the association with endocarditis, blood cultures should be drawn in all patients with endogenous endophthalmitis prior to the institution of broad spectrum antibiotics. Hepatitis C and HIV coinfection is also common and too should be evaluated in these cases. The prognosis in* Rhodotorula* endogenous endophthalmitis is grave, so increased investment in prevention of IDU is critical.

## Figures and Tables

**Table 1:** * Rhodotorula* endogenous endophthalmitis cases to present.

Year	Country	Age/sex	Coinfection	History of IDU	Affected eye	Treatment	Visual outcome
2001 [4]	Italy	27/M	Hepatitis C	Positive	Right	IA + oral ketoconazole	Uncertain light perception
2002 [5]	Canada	26/M	HIV	Positive	Right	IA + SA	Enucleation
2011^*∗*^	USA	21/M	Hepatitis C	Positive	Left	IA + oral posaconazole	No light perception

^*∗*^The current case presented

HIV: Human Immunodeficiency Virus

IA: intravitreal amphotericin B, SA: systemic amphotericin B.
